# Identifikation rheumatologischer Gesundheits-Apps im Apple App Store mit der Methode der „semiautomatischen retrospektiven App Store-Analyse“: Eine longitudinale Betrachtung

**DOI:** 10.1007/s00393-021-01099-9

**Published:** 2021-10-11

**Authors:** J. G. Richter, G. Chehab, U. Kiltz, A. Becker, U. von Jan, U.-V. Albrecht, M. Schneider, C. Specker

**Affiliations:** 1 Poliklinik und Funktionsbereich Rheumatologie & Hiller-Forschungszentrum Rheumatologie, Medizinische Fakultät, Heinrich-Heine-Universität Düsseldorf, Universitätsklinikum Düsseldorf, Moorenstr. 5, 40225 Düsseldorf, Deutschland; 2 Rheumazentrum Ruhrgebiet, Ruhr-Universität Bochum, Herne, Deutschland; 3 Ortenau Klinikum Offenburg-Kehl, Offenburg, Deutschland; 4 Peter L. Reichertz Institut für Medizinische Informatik der TU Braunschweig und der Medizinischen Hochschule Hannover, Medizinische Hochschule Hannover, Carl-Neuberg-Str. 1, 30625 Hannover, Deutschland; 5 AG 4 – Digitale Medizin, Medizinische Fakultät OWL, Universität Bielefeld, Universitätsstr. 25, 33615 Bielefeld, Deutschland; 6 Klinik für Rheumatologie & Klinische Immunologie, Evangelisches Krankenhaus, Kliniken Essen-Mitte, Essen, Deutschland

**Keywords:** Digitale Gesundheitsanwendungen, Digitale Rheumatologie, eHealth, Gütesiegel, Qualitätskriterien, Digital health applications, Digital rheumatology, ehealth, Quality seals, Quality criteria

## Abstract

**Hintergrund:**

Die App Stores von Apple und Google bieten eine Vielzahl von Gesundheits-Apps an. Das Auffinden qualitativ hochwertiger Apps ist immer noch eine Herausforderung.

**Fragestellung:**

Lassen sich unter Anwendung der SARASA(„semiautomated retrospective App Store analysis“)-Methode für das Fachgebiet Rheumatologie deutschsprachige Apps identifizieren?

**Material und Methode:**

SARASA ist eine Methode zur teilautomatisierten Auswahl und Charakterisierung von App Store-gelisteten Apps nach formalen Kriterien. Nach der ersten Anwendung in 02/2018 wurde SARASA 02/2020 erneut auf den Apple App Store angewendet.

**Ergebnisse:**

In 02/2018 konnten für Apps in den Store-Kategorien „Medizin“ oder „Gesundheit und Fitness“ Metadaten zu 103.046 Apps und bei einer erneuten Erhebung in 02/2020 Daten zu 94.735 Apps über das deutsche Frontend des Apple App Stores ausgelesen werden. Im Jahr 2018 wurden nach Anwendung der Suchbegriffe 59 Apps mit einer deutschsprachigen Beschreibung für das Fachgebiet Rheumatologie identifiziert, 2020 waren dies 53 Apps, die jeweils manuell weiter überprüft wurden; 2018 waren noch mehr der gefundenen Apps für Patienten als für Ärzte vorgesehen, dies war 2020 ausgeglichener. Zudem zeigte sich, dass bei bestimmten Krankheitsbildern von den App-Entwicklern keine Bearbeitungen erfolgten. Die prozentuale Verteilung von Treffern nach Suchbegriffen zeigte im Vergleich von 2018 zu 2020 große Schwankungen.

**Diskussion:**

Die SARASA-Methode stellt ein hilfreiches Werkzeug dar, um Gesundheits-Apps teilautomatisiert zu identifizieren, die vordefinierten, formalen Kriterien entsprechen. Die inhaltliche Qualität muss anschließend manuell überprüft werden. Weiterentwicklungen der SARASA-Methode und die weitere Konsentierung und Standardisierung von Qualitätskriterien sind sinnvoll. Qualitätskriterien sollten beim Angebot von Gesundheits-Apps in den App-Stores berücksichtigt werden.

## Abstract (en)

**Background:**

The Apple and Google app stores offer a wide range of health apps. It is still a challenge to find valuable and qualified apps.

**Objective:**

Can German language apps be identified using the “semiautomated retrospective app store analysis” (SARASA) method for the field of rheumatology?

**Material and method:**

The SARASA is a semiautomated method to select and characterize apps listed in the app store. After the first application in February 2018 SARASA was applied again to the Apple app store in February 2020.

**Results:**

In February 2018 it was possible to acquire metadata for 103,046 apps and in February 2020 data for 94,735 apps that were listed in the category “health and fitness” or “medicine” in Apple’s app store frontend for Germany. After applying the search terms 59 apps with a German language app description were identified for the field of rheumatology in 2018 and 53 apps in 2020. For these, more detailed manual reviews seem worthwhile. In 2018, the apps found were more likely to address patients than physicians and this was more balanced in 2020. In addition, it became apparent that for certain diseases there was no app developer activity. The percentage breakdown of matches by search term revealed substantial fluctuations in the app market when comparing 2018 to 2020.

**Discussion:**

The SARASA method provides a useful tool to identify apps from app stores that meet predefined, formal criteria. Subsequent manual checks of the quality of the contents are still necessary. Further development of the SARASA method and consensus and standardization of quality criteria are worthwhile. Quality criteria should be considered for offers of mobile health apps in app stores.

## Hintergrund

App Stores wie die von Apple und Google bieten eine enorme Vielzahl gesundheitsbezogener Apps zum Direktbezug durch die Verbraucher an. Bei Fehlen einer klinischen oder regulatorischen Bewertung resultiert eine heterogene Qualität [1]. Diese Situation wird dadurch gefördert, dass die Entwicklung von (Gesundheits‑)Apps einfacher und kostengünstiger geworden ist, z. B. durch die Nutzung von sog. App-Entwicklungstoolkits [2, 3]. Niedrige Marktbarrieren ziehen unterschiedlich qualifizierte Unternehmen mit variablen Geschäftsmotivationen an, um in den profitablen Gesundheitsmarkt einzusteigen [2–4].

Bedeutsam ist in diesem Kontext das durch das Digitale-Versorgung-Gesetz (DVG) seit 2020 entstehende „digitale Gesundheitsanwendungen(DiGA)-Verzeichnis“. Dieses beim Bundesinstitut für Arzneimittel und Medizinprodukte (BfArM) geführte Verzeichnis ist als ein Mittel zur Regulierung für den deutschen Markt zu verstehen [5]. In Deutschland können DiGAs gemäß DVG verordnet und auch bei Eigenbeantragung durch den Versicherten von den gesetzlichen Krankenkassen erstattet werden. Entsprechende organisatorische Strukturen wurden implementiert, weitere, detaillierte Ausgestaltungen sind in Vorbereitung. DiGAs sollen, um langfristig in das DiGA-Verzeichnis beim BfArM aufgenommen werden zu können, im klinischen Kontext – auch auf ihren Nutzen – geprüft werden [5, 6], einzelne Ergebnisse zur Validität der mit Gesundheits-Apps erhobenen Daten liegen für die Rheumatologie vor [7, 8].

Der Anwendung von Gesundheits-Apps werden vielversprechende Erfolgsaussichten zugeschrieben [7, 9]. Postuliert wird ein großes Potenzial, die Gesundheit und das Patientenmanagement zu verbessern, aber es besteht auch hier eine erhebliche Heterogenität, z. B. hinsichtlich ihrer Sicherheit und Qualität [2, 3]. Zu den diskutierten Qualitätsthemen zählen beispielsweise zweifelhafte App-Inhalte, der Verlust der Privatsphäre in Verbindung mit Weitergabe der erfassten Patientendaten sowie die fehlenden Daten zur Wirksamkeit einer Gesundheits-App [1, 3]. Gründe dafür, warum geringe App-Qualität durchaus üblich ist und zudem auch weithin toleriert wird und warum ihre Regelung so schwierig ist, wurden publiziert [3, 10]. Dazu zählen Gründe auf der Entwickler-, der Anwender-, der regulatorischen und der ethischen Seite. Beispiele sind die hohe Anzahl von Apps, ein geringes Bewusstsein für Qualität und Qualitätskriterien, die Kultur des sog. „minimal viable products“ bei Veröffentlichung der Apps, niedrige Markteintrittsbarrieren, der häufig bestehende Mangel formaler Evaluationen und des Monitorings der Nutzung sowie der noch in Entwicklung befindliche Konsens der beteiligten Stakeholder über den „Grad der akzeptablen Sicherheit“ [3, 10].

Auch wenn Forschung zur Identifizierung, Charakterisierung und Bewertung von Gesundheits-Apps erfolgt, ist es immer noch eine Herausforderung, qualitativ hochwertige Apps zu finden und sich nicht nur von subjektiven, nicht standardisierten und semiquantitativen Beurteilungen mit „Sternen“ anderer Nutzer im App Store leiten zu lassen. Um rheumatologisch Interessierte bei der Suche nach qualitativ höherwertigen Apps zu unterstützen, untersuchten wir, wie die von Albrecht et al. publizierte SARASA(„semiautomated retrospective App Store analysis“)-Methode rheumatologische Apps [11] identifizieren kann. Wir prüften, ob diese – über ggf. notwendige Ergänzungen – mit Qualitätsindikatoren annotiert und somit für die Versorgung der Patienten als vorteilhafte Apps hervorgehoben werden können.

## Material und Methode

Die „semiautomated retrospective App Store analysis“ (SARASA) ist eine mehrstufige Methode zur Auswahl und Charakterisierung von in einem App Store gelisteten Apps, die exemplarisch für den App Store von Apple entwickelt und von Albrecht et al. beschrieben wurde [11], prinzipiell aber auch auf andere Stores anwendbar wäre.

Im ersten SARASA-Schritt werden die Metadaten der in den gewünschten Apple App Store-Kategorien (z. B. „Medizin“ und/oder „Gesundheit und Fitness“) enthaltenen Apps über deren Listung auf den länderspezifischen Webseiten der jeweiligen Store-Kategorien und die Verwendung des „iTunes Search APIs“ [12] skriptbasiert ausgelesen. Dazu gehören u. a. die App-Namen, Angaben zu den Herstellern, Links zur Website des Herstellers, die Store-Beschreibungstexte zu den Apps, notwendige Betriebssystemversion, Kosten, Datum der Bereitstellung bzw. letzten Aktualisierung oder auch Nutzerbewertungen [11]. Schritt 2 setzt sich mit der Zusammenstellung der Filterkriterien für die gewonnenen Daten auseinander. Zur Analyse der textuellen Informationen – insbesondere der Store-Beschreibungstexte der Apps – werden für die Suche geeignete, fachbezogene Stichworte definiert. Hierzu werden idealerweise sog. reguläre Ausdrücke in Perl-Notation [13] verwendet, um nötigenfalls verschiedene Wortformen bzw. Wortkombinationen erfassen oder vermeiden zu können [14]. Zudem ist es möglich, Rankingkriterien auf Basis der weiteren Metadaten festzulegen (z. B. das Vorhandensein oder Fehlen bestimmter Herstellerangaben oder auch, ob eine App kostenfrei bzw. kostenpflichtig ist) und diesen Wichtungen zuzuordnen. Dies kann insbesondere bei einer zu erwartenden großen Trefferanzahl im späteren Verlauf helfen, Apps, die diesen Rankingkriterien besonders gut entsprechen, in der Ergebnisliste prominenter abzubilden [11]. Im dritten SARASA-Schritt werden die zuvor definierten Suchstichworte auf die verfügbaren App-Daten angewendet, um eine Auswahl von Apps aus der Datenbank zu extrahieren [11]. Anschließend wird die Ergebnisliste manuell validiert sowie, wenn gewünscht, kategorisiert, und es werden (optional und dann automatisiert) die zuvor definierten Ranking-Kriterien angewendet [11]. Abschließend steht eine Liste von Apps mit den begleitenden Metadaten zur Verfügung, die für beliebige weitere Auswertungen herangezogen werden kann [11].

Im Februar 2018 und erneut im Februar 2020 wurde die SARASA-Methode auf den Apple App Store auf Apps angewendet, für die in den Metadaten in der sog. primären oder sekundären Kategorie „Gesundheit und Fitness“ und „Medizin“ angegeben war (vom Hersteller festgelegt) und die somit auf den oben genannten Webseiten gelistet waren. In einem zweiten Schritt wurden die Ergebnisse mit von den Autoren gewählten rheumatologischen Suchbegriffen systematisch eingegrenzt. Dazu wurden 2018 und 2020 die identischen Suchbegriffe (ohne Berücksichtigung der Groß‑/Kleinschreibung) genutzt: arthritis, spondyl, uveitis, collagenos, kollagenos, lupus, vasculitis, vaskulitis, arteriitis, granulomatose, sklero, sclero, myositis, sjögren und rheumatolog. Auf das optionale Festlegen und Anwenden von Rankingkriterien wurde hier verzichtet. Anschließend erfolgte eine manuelle Überprüfung der identifizierten deutschsprachigen Apps durch die Autoren. Die Gesundheits-Apps wurden strukturiert erfasst und nach von den Autoren konsentierten Zielgruppen ärztliches oder medizinisches Fachpersonal und Patienten kategorisiert. Darüber hinaus wurde untersucht, ob zwischen 2018 und 2020 eine Zu- oder Abnahme der Anzahl von deutsch- und englischsprachigen Apps zu verzeichnen war. Diese Betrachtung erfolgte auch bezüglich der einzelnen Krankheitsgruppen bzw. Diagnosen gemäß den genutzten Suchbegriffen.

Das DiGA-Verzeichnis beim BfArM (https://diga.bfarm.de/de/verzeichnis) wurde am 15.04.2021 daraufhin überprüft, ob eine oder mehrere der in 2018 bzw. 2020 mittels SARASA und den anschließend vorgenommenen manuellen Überprüfungsprozessen der Autoren gefundenen Apps gelistet werden.

## Ergebnisse

### Identifikation rheumatologischer Apps mit der SARASA-Methode

Mit der SARASA-Methode wurden im Februar 2018 103.046 Treffer in der sog. primären oder sekundären Kategorie „Gesundheit und Fitness“ und „Medizin“ erzielt, davon 10.935 mit deutsch- (DE) und 70.668 mit englischsprachiger (EN) Beschreibung. Nach Anwendung der im Methodenteil beschriebenen rheumatologischen Suchbegriffe wurden 574 EN und 59 DE Apps identifiziert (vgl. Tab. 1).

**Table 1:** 

	2018		2020		Differenz zwischen 2018 und 2020		Prozentuale Veränderung zwischen 2018 und 2020	
	Treffer (EN)	Treffer (DE)	Treffer (EN)	Treffer (DE)	EN	DE	EN	DE
	70.668	10.935	63.769	9639	−6899	−1296	−10	−12
*Suchbegriffe*								
*arthritis	284	17	240	14	−44	−3	−16	−18
Spondyl*	25	6	33	7	8	1	32	17
Uveitis	8	1	7	0	−1	−1	−13	−100
Collagenos	0	0	0	0	0	0	Nb	Nb
Kollagenos	0	0	0	0	0	0	Nb	Nb
Lupus	44	5	40	1	−4	−4	−9	−80
Vasculitis	11	0	10	0	−1	0	−9	Nb
Vaskulitis	0	0	0	0	0	0	Nb	Nb
*arteriitis	0	0	0	0	0	0	Nb	Nb
Granulomatos*	2	1	1	1	−1	0	−50	0
Sklero*	4	31	4	29	0	−2	0	−7
Sclero*	187	1	154	1	−33	0	−18	0
Myositis	5	0	8	0	3	0	60	Nb
Sjögren	7	0	4	0	−3	0	−43	Nb
Rheumatolog	108	6	84	11	−24	5	−22	83
Summe Apps^a^	574	59	484	53	−90	−6	−16	−10

*Nb* mathematisch nicht berechenbar

^a^Doppelnennung werden nicht gezählt

Im Februar 2020 zeigten sich mit der Methode 94.735 Treffer in den Kategorien „Gesundheit und Fitness“ und „Medizin“, davon 9639 mit deutsch- und 63.769 mit englischsprachiger Beschreibung. Nach Anwendung derselben Suchbegriffe wurden im Vergleich zu Februar 2018 noch 484 EN und 53 DE Apps, also 90 EN Apps (−16 %) und 6 DE Apps (−10 %) weniger identifiziert (vgl. Tab. 1). Die Tab. 1 stellt zudem die Differenzen bzw. die prozentualen Veränderungen nach Stichworten getrennt dar. Dabei war der größte prozentuale Zuwachs im Bereich der Spondyloarthritiden nachzuweisen. Beispielsweise für den systemischen Lupus (SLE) zeigte sich, dass eine App, die ein elektronisches Patiententagebuch darstellt (zur Verfügung gestellt seitens der pharmazeutischen Industrie), 2018 und 2020 aufgelistet wird. Drei Apps, die ebenfalls elektronische Patiententagebücher sind, aber nicht SLE-spezifisch waren, wurden zwar 2018, nicht mehr aber 2020 unter dem Stichwort Lupus aufgeführt. Die Suche nach deutschsprachigen, rheumatologischen Apps identifizierte nach 2 Jahren 6 „neue“ Apps, und 7 waren nicht mehr im App Store verfügbar.

In der Gesamtauswertung zeigte sich zudem, dass einige Krankheitsbilder bzw. Krankheitsgruppen von App-Entwicklern auch im Verlauf von 2018 bis 2020 nicht bearbeitet werden (z. B. Riesenzellarteriitis und Myositis) und anhand der Anzahl aufgelisteter Apps auch ein Rückgang der Interessenlage an einzelnen Krankheitsbildern bzw. Krankheitsgruppen zu verzeichnen ist (vgl. Tab. 1).

Bereits bei der ersten Anwendung der Methode im Jahr 2018 wurde für die Autoren schnell deutlich, dass die Suchbegriffe Sklero* und Sclero* auch die Apps identifiziert, die im Kontext der neurologischen Diagnose „multiple Sklerose“ zum Einsatz kommen können, also eine Art Cross-Referenzierung haben. Ein sinnvoller Ersatz dieser Suchbegriffe konnte von den Autoren auch für den Einsatz im Jahr 2020 nicht gefunden werden, da die aktuelle rheumatologische Diagnosenomenklatur weiter „systemische Sklerose“ lautet.

### Manuelle Überprüfung der mit SARASA identifizierten Apps

Die manuelle Überprüfung der im Jahr 2018 mit SARASA und den vorgegebenen Suchbegriffen identifizierten 59 Apps ergab 19 DE Apps als potenziell relevant für die Rheumatologie. Diese erfüllen die durch die SARASA-Methode vorgegebenen, formalen Suchkriterien, z. B. vom Hersteller bereitgestellte App-Beschreibungen. Von diesen 19 Apps richteten sich 11 an Patienten (2 für rheumatoide Arthritis, 4 für Spondylarthritis, 1 für systemischen Lupus erythematodes, 3 für Patienten mit chronischen Schmerzen und 1 für Patienten ohne spezifische Krankheitszuordnung [Rhekiss Register-App]) und 5 an ärztlich Tätige bzw. medizinisches Fachpersonal (vgl. Tab. 2). Drei Apps waren nicht bewertbar, da sie bei der manuellen Prüfung im App Store nicht mehr abrufbar waren.

**Table 2:** 

Name der App	Diagnose	Verfügbar		Durchschnittliche Sterne-Bewertung (Anzahl abgegebener Bewertungen)^a^		Medizinisches Fachpersonal	Patient	App Store-Link 2020, dort sind auch Herstellerangaben einsehbar
		2018	2020	2018	2020			
Rheuma AKTIV – Hilfreiche Tipps bei Rheumatoider Arthritis	RA	X	–	5 (2)	–	–	Elektronisches Patiententagebuch	Nicht verfügbar
RheumaLive	RA	X	X	NA	NA	–	Elektronisches Patiententagebuch	https://apps.apple.com/de/app/rheumalive/id987847035
RheumaBuddy	RA	–	X	–	NA	–	Elektronisches Patiententagebuch & Community	https://apps.apple.com/de/app/rheumabuddy/id920743331
Rheumatoide Arthritis i‑pocketcards	RA	X	–	3 (5)	–	„Aktuelle“ Diagnostik- und Therapieempfehlungen	–	Nicht verfügbar
AQUILA-App	SpA	X	X	NA	3 (1)	–	Elektronisches Patiententagebuch	https://apps.apple.com/de/app/aquila-app/id1239787078
ASAS App	SpA	X	X	NA	NA	Berechnung von Scores, ASAS-Klassifikationskriterien	–	https://apps.apple.com/de/app/asas-app/id650291528
AxSpALive	SpA	X	X	2 (1)	NA	–	Elektronisches Patiententagebuch	https://apps.apple.com/de/app/axspalive/id1076252165
PsALive	SpA	X	X	5 (1)	5 (1)	–	Elektronisches Patiententagebuch	https://apps.apple.com/de/app/psalive/id1164383329
PSORIapp	SpA	X	–	NA	–	–	Elektronisches Patiententagebuch, Informationen zu Bewegung und Ernährung	Nicht verfügbar
LupusLog	Lupus erythematodes	X	X	NA	NA	–	Elektronisches Patiententagebuch	https://apps.apple.com/de/app/lupuslog/id983935408
Systemischer Lupus erythematodes	Lupus erythematodes	X	–	NA	–	x	–	Nicht verfügbar
Sklerodermie	Sklerodermie	X^b^	–	NA	–	–	–	Nicht verfügbar
ANCA-assoziierte Vaskulitiden	Vaskulitis	X	X	NA	NA	Nur für medizinische Fachkreise. DocCheck-Login erforderlich	–	https://apps.apple.com/de/app/anca-assoziierte-vaskulitiden/id907431991
Arzneimittel pocket 2018	–	X	X	3 (148)	3 (189)	Nachschlagewerk Medikation	–	https://apps.apple.com/de/app/arzneimittel-pocket-2018/id989086879
Laborwerte 3 bzw. 4	–	X^b^	X	NA	NA	Nachschlagewerk Labor	(Nachschlagewerk Labor)	https://apps.apple.com/de/app/laborwerte-4/id1390397435
Rheumatologie visuell	–	X	X	NA	NA	Bildbefunde	–	https://apps.apple.com/de/app/rheumatologie-visuell/id1139133820
Deutsches GesundheitsPortal	–	–	X	–	5 (1)	Literaturdatenbank, aktuelle Studien kurzgefasst	–	https://apps.apple.com/de/app/deutsches-gesundheitsportal/id1435062119
Manage My Pain	„Schmerzpatienten“	–	X	–	NA	–	Elektronisches Patiententagebuch	https://apps.apple.com/de/app/manage-my-pain/id1444320523
Schmerztagebuch & Community CatchMyPain	„Schmerzpatienten“	X	–	4 (54)	–	–	Elektronisches Patiententagebuch & Community	Nicht verfügbar
Schmerztagebuch & Community CatchMyPain mit Medikamentenerfassung	„Schmerzpatienten“	X	–	4 (46)	–	–	Elektronisches Patiententagebuch & Community	Nicht verfügbar
Schmerztagebuch & Community CatchMyPain PRO	„Schmerzpatienten“	X	–	4,5 (51)	–	–	Elektronisches Patiententagebuch & Community	Nicht verfügbar
Rheuma Schweiz Education	–	–	X	–	NA	Edukation B2B	–	https://apps.apple.com/de/app/rheuma-schweiz-education/id911358609
RheumaGuide	–	–	X	–	NA	RheumaGuide zur Diagnostik und Therapie der Erkrankungen	–	https://apps.apple.com/de/app/rheumaguide/id744929909
Rheuma-VOR	–	–	X	–	NA	Diagnoseunterstützung	Diagnoseunterstützung	https://apps.apple.com/de/app/rheuma-vor/id1436442062
Rhekiss	Verschiedene	X	X	NA	1 (1)	–	Elektronisches Dokumentationssystem, Register	https://apps.apple.com/de/app/rhekiss/id1219957600
BeActive	?	X^b^	–	5 (5)	–	–	–	Nicht verfügbar

*NA* fehlende Bewertungen im App Store, *RA* rheumatoide Arthritis, *SpA* axiale Spondyloarthritis, *ASAS* Assessment of Spondyloarthritis International Society, *ANCA* antineutrophile zytoplasmatische Antikörper

^a^Die Zahlen beruhen auf allen zur jeweiligen App abgegebenen Bewertungen, nicht nur auf den Daten für die zum Auslesezeitpunkt aktuellen Versionen, ^b^Link bei manueller Prüfung nicht mehr zugänglich

Die manuelle Überprüfung der im Jahr 2020 mittels SARASA und Suchbegriffen identifizierten 53 Apps wies 17 DE Apps als potenziell relevant für die Rheumatologie aus. Auch diese Apps erfüllen die durch die Methode vorgegebenen Mindestkriterien (für Details vgl. auch Tab. 2).

Die Tab. 2 listet die 2018 und/oder 2020 identifizierten deutschsprachigen Apps namentlich auf. Zudem stellt die Tab. 2 die 2020 identifizierten App Store-Links zur Verfügung. Bewertungen von rheumatologischen Apps werden im App Store eher selten vergeben.

Keine der in den Tabellen genannten Apps war zum 15.04.2021 im DiGA-Verzeichnis des BfArM gelistet.

Darüber hinaus wurden im Jahr 2018 5 und im Jahr 2020 weitere 7 Apps identifiziert, die übergeordnet für die nichtfachspezifische respektive studentische Ausbildung einsetzbar wären. Dazu zählen Apps, die beispielsweise die Sonographie oder die Anatomie lehren wollen (Tab. 3). Eine inhaltliche Prüfung dieser Apps erfolgte nicht.

**Table 3:** 

Name der App	2018	2020	Durchschnittliche Sterne-Bewertung (Anzahl abgegebener Bewertungen)^a^		App Store-Link 2020, dort sind auch Herstellerangaben einsehbar
			2018	2020	
Physiologie & Pathologie	–	X	–	4,5 (42)	https://apps.apple.com/de/app/physiologie-pathologie/id1477240784
Physiology Animations	X	X	5 (1)	2,5 (7)	https://apps.apple.com/de/app/physiology-animations/id715136002
HipBiomechanicsApp	–	X	–	NA	https://apps.apple.com/de/app/hipbiomechanicsapp/id1390242983
Deepscope Ultrasound Simulator	–	X	–	NA	https://apps.apple.com/de/app/deepscope-ultrasound-simulator/id1481238908
Patient Education Institute	X	X	NA	5 (1)	https://apps.apple.com/de/app/patient-education-institute/id883742086
VEPRO WEBstudio	X	X	NA	NA	https://apps.apple.com/de/app/vepro-webstudio/id1314301300
Muscle Premium	X	X	4,5 (49)	4 (60)	https://apps.apple.com/de/app/muscle-premium/id504001448
CME Allgemeinmedizin Schett	X	–	NA	–	2020 nicht verfügbar

*NA* fehlende Bewertungen im App Store

^a^Die Zahlen beruhen auf allen zur jeweiligen App abgegebenen Bewertungen, nicht nur auf den Daten für die zum Auslesezeitpunkt aktuellen Versionen

## Diskussion

SARASA ist eine Methode zur teilautomatisierten Auswahl und Charakterisierung von in einem App Store gelisteten Apps gemäß formaler Kriterien [11]. Nach Kenntnis der Autoren beschreibt die vorliegende Publikation erstmalig die Anwendung der SARASA-Methode im Fachgebiet der Rheumatologie und stellt ihre vergleichende Anwendung im Abstand von 2 Jahren dar. Die Zahl der mittels definierter Qualitätskriterien herausgefilterten Apps war sowohl 2018 als auch 2020 mit weniger als 20 deutschsprachigen Apps überschaubar. Die prozentualen Veränderungen bei den einzelnen Stichworten verdeutlichen einerseits die Fluktuationen am App-Markt, könnten aber auch darauf hinweisen, dass die von den Herstellern zur Verfügung gestellten Metadaten der Apps ggf. adaptiert wurden und damit bei der Analyse nicht mehr mit ausgegeben werden. Albrecht et al. identifizierten 2018 in einem vergleichbaren Ansatz *n* = 335 kardiologische deutschsprachige Apps, die dann durch Studierende weiter evaluiert wurden [11, 15].

Das große Angebot von Apps in den App Stores, die schnellen Entwicklungen digitaler Gesundheitsanwendungen sowie die inhaltliche und technische Qualität, die auch die publizierten Risiken der Verwendung von Gesundheits-Apps beinhaltet, machen kontinuierliche Beurteilungen notwendig [1, 3, 16]. Albrecht et al. forderten bereits 2018 ein „gesetzlich verpflichtendes, standardisiertes Berichtswesen“ für Apps [17]. Mit dem Digitale-Versorgung-Gesetz und dem daraufhin implementierten DiGA-Verzeichnis des BfArM wurden zwar wesentliche Schritte in diese Richtung vollzogen [5], mit Stand 15.04.2021 war aber noch keine der mit der SARASA-Methode identifizierten Apps im DiGA-Verzeichnis zu finden. Aus vielfältigen Gründen werden sich nicht alle App-Hersteller dem aufwendigen und kostenintensiven DiGA-Prozess stellen wollen und können und dennoch ihre Apps in den Stores anbieten [6]. Erste Hersteller potenzieller DiGAs haben bereits ihre Anträge auf Zulassung zurückgezogen, und einige Anträge wurden vom BfArM auch negativ beschieden [18]. Zu welchen Fach- bzw. Anwendungsgebieten diese gehören, ist (zumindest bis zur Erstellung dieser Publikation) nicht publik. Darüber hinaus mag die fehlende Auffindbarkeit der Apps im DiGA-Verzeichnis auch Ausdruck der hohen Fluktuation von Apps und ihrer Hersteller sein. Bereits bei unserer zeitnahen manuellen Rezension waren einzelne der mit SARASA identifizierten Apps veraltet (beispielsweise 2020 die RheumaLive App, die in die RheCord App überführt wurde).

Kontinuierliche Beurteilungen der Apps sind notwendig

Die Forschung zur Identifizierung, Charakterisierung und Bewertung gesundheitsbezogener Apps wird aber nicht nur durch die Dynamik digitaler Gesundheitsanwendungen, sondern auch durch eine große Heterogenität von Bewertungskriterien erschwert. Daraus resultierten Forderungen nach einem möglichst einfach (automatisiert) einzusetzenden, standardisierten Bewertungstool zur Qualitätsprüfung, auch um relevante und zuverlässige Apps besser identifizieren zu können [19, 20]. Gütesiegel wurden diskutiert, eines wurde beispielsweise von einer Initiative aus dem Bereich der Diabetologie (DiaDigital https://www.diabetesde.org/diadigital) implementiert [21].

Die von einigen App-Herstellern genutzten CE-Kennzeichnungen mit Identifikationsnummern dokumentieren lediglich die Einhaltung gesetzlicher Mindestanforderungen der Europäischen Union und weisen auf die Einbindung einer sog. „benannten Stelle“ hin. Die CE-Kennzeichnungen stellen rechtlich und v. a. inhaltlich aber kein Gütekriterium dar. Es existieren aber Bemühungen der Europäischen Kommission zur Entwicklung einer technischen Spezifikation von Qualitäts- und Reliabilitätsanforderungen für „Gesundheits- und Wellness-Apps“. Die Arbeiten werden vom Europäischen Komitee für Normung (CEN) geleitet, das mit der Internationalen Organisation für Normung (ISO), der Internationalen Elektrotechnischen Kommission (IEC) und dem Europäischen Komitee für elektrotechnische Normung (CENELEC) zusammenarbeitet [1]. Von der EULAR (European League Against Rheumatism) wurden inzwischen sog. „points to consider“ publiziert, die bei der Entwicklung, Bewertung und Implementierung mobiler Gesundheitsanwendungen zur Unterstützung des Selbstmanagements von Menschen mit rheumatischen und muskuloskeletalen Erkrankungen zu beachten sind [22].

Portale wie beispielsweise die sog. „Weiße Liste“ verzeichnen digitale Gesundheitsanwendungen, die als Medizinprodukt zertifiziert sind und wollen den User mit zuverlässigen Informationen bei der Auswahl unterstützen [23]. Die Pflege solcher Seiten ist aber aufwendig. Bislang (Stand 20.04.2021) ist dort eine einzige rheumatologische App gelistet, die aus 3 der von uns sowohl 2018 als auch 2020 mit SARASA identifizierten Apps durch Fusionierung entstanden ist. Das Zentralinstitut für die kassenärztliche Versorgung in der Bundesrepublik Deutschland stellt seit Ende 2020 die Website „KVAppRadar“ zur Verfügung, die zunächst im Testbetrieb Ärzten und Psychotherapeuten bzw. zu einem späteren Zeitpunkt auch Patienten die Möglichkeit geben soll, sich über Gesundheits-Apps zu informieren [24]. Ein weiteres Angebot zu DiGAs findet sich in dem sog. „DiGA Monitor“, der von einer Privatperson angeboten wird und auch aktuelle Preise der DiGAs auflistet [25]. Weitere Verbände wie der Verband der forschenden Pharma-Unternehmen in Deutschland stellen Interessierten Informationen zu DiGAs u. a. in Form der „DiGA Watchlist“ zur Verfügung [26].

Verschiedene Analysetools, wie z. B. die entwickelte Mobile App Rating Scale (MARS), sollen ebenfalls helfen, relevante, qualitativ hochwertige Apps zu identifizieren [2, 20, 27]. Für einen Teil der mit SARASA identifizierten Apps sind entsprechende MARS-Evaluationen publiziert [2]. Zur Praktikabilität und Anwendbarkeit in der klinischen Versorgung der anhand der SARASA-Methode und der von uns durch manuelle Überprüfung identifizierten Apps liegen nur einzelne Erfahrungen aus Proof-of-Concept-Studien vor [7, 8].

Bevor zeitaufwendige inhaltliche Analysen, z. B. inhaltsbasierte Auswertungen zur Beurteilung der Qualität einzelner Apps, erfolgen, scheint es – auch aus ökonomischen Gründen – sinnvoll, eine standardisierte valide Vorauswahl zu treffen und nicht ein rein händisches Durchsuchen der App Stores mittels Suchbegriffen anzustreben. So liefert die Anwendung der SARASA-Methode semiautomatisiert Apps, bei denen zumindest auf die vom Hersteller zur Verfügung gestellten App-Beschreibungen und andere Metadaten geprüft wurde. Die verpflichtende Einführung weiterer Metadaten zu den Gesundheits-Apps in App Stores, wie sie z. B. bei PubMed durch die sog. MeSH(Medical Subject Headings)-Terms existieren, könnte ggf. auch das Problem nicht eineindeutig zu bestimmender Suchbegriffe lösen helfen. Das „Training“ der der SARASA-Methode zugrunde liegenden Algorithmen anhand dieser Evaluationen könnte möglicherweise zur weiteren Qualitätsoptimierung der Methode beitragen.

## Fazit für die Praxis

- Zusammengefasst stellt die SARASA(„semiautomated retrospective App Store analysis“)-Methode auch für die Rheumatologie ein Werkzeug dar, um Apps aus App Stores zu identifizieren, die vordefinierten, formalen Kriterien entsprechen.
- Die resultierende App-Auswahl mit begleitenden zusammenfassenden Beschreibungen kann und sollte für weitere Auswertungen, z. B. inhaltsbasierte Auswertungen zur Beurteilung der App-Qualität, verwendet werden.
- Aufgrund der limitierten Trefferzahl ist unter Einbeziehung der Patienten(‑Organisationen) eine regelmäßige Veröffentlichung von Bewertungen denkbar, die Interessierten als Ergänzung der App Stores zur Verfügung gestellt werden kann.
- Qualitätskriterien für Gesundheits-Apps müssen noch immer weiterentwickelt und international standardisiert werden, um auch Fachdisziplin-übergreifend und im internationalen Markt vergleichbare Bewertungen zu ermöglichen.
